# Anti-oxidant and Anti-inflammatory Effects of Aquatic Exercise in Allergic Airway Inflammation in Mice

**DOI:** 10.3389/fphys.2019.01227

**Published:** 2019-09-24

**Authors:** Boae Lee, Yeonye Kim, Young Mi Kim, Jaehoon Jung, Taehyung Kim, Sang-Yull Lee, Yong-Il Shin, Ji Hyeon Ryu

**Affiliations:** ^1^Department of Rehabilitation Medicine, School of Medicine, Pusan National University, Busan, South Korea; ^2^Research Institute for Convergence of Biomedical Science and Technology, Pusan National University Yangsan Hospital, Yangsan, South Korea; ^3^Department of Biochemistry, School of Medicine, Pusan National University, Busan, South Korea

**Keywords:** allergic airway inflammation, aquatic exercise, swimming, oxidative stress, inflammation

## Abstract

Oxidative stress and inflammation are key pathways responsible for the pathogenesis of asthma. Aquatic exercise (AE) has been proven to elicit a variety of biological activities such as anti-oxidant and anti-inflammatory effects. However, although proper forms of AE provide beneficial health effects, incorrect forms and types of AE are potentially injurious to health. Several studies have investigated AE, but the relationship between types of AE and asthma has not been fully elucidated. This study evaluated the effects of two types of AE according to resistance on ovalbumin (OVA)-induced allergic airway inflammation in mice. BALB/c mice were subjected to OVA sensitization and challenge, and then to different types of AE including, walking and swimming, in a pool filled with water to a height of 2.5 and 13 cm for 30 min, respectively. AE reduced OVA-induced eosinophilic inflammation, airway hyperresponsiveness, and serum immunoglobulin E level. AE significantly inhibited increases in interleukin (IL)-4, IL-5, IL-13, histamine, leukotriene D4, and tryptase levels in bronchoalveolar lavage fluid (BALF). AE also effectively suppressed mucus formation, lung fibrosis, and hypertrophy of airway smooth muscle within the lung tissues. This exercise markedly reduced the levels of malondialdehyde while increased glutathione and superoxide dismutase (SOD) activity in lung tissues. Furthermore, AE significantly decreased tumor necrosis factor-α, IL-6 levels, and prostaglandin E2 production in BALF. The inhibitory effects of swimming on the levels of biomarkers related to oxidative stress and inflammation were greater than that of walking. These effects may have occurred through upregulation of NF-E2-related factor 2/heme oxygenase-1 signaling and suppression of mitogen-activated protein kinase/nuclear factor-κB pathway. Cumulative results from this study suggest that AE might be beneficial in mitigating the levels of biomarkers related to oxidative stress and inflammation. Thus, this therapy represents a crucial non-pharmacological intervention for treatments of allergic airway inflammation.

## Introduction

Asthma, one of the most common respiratory diseases, is caused by combinations of multiple environmental factors. Clinical features include recurrent coughing, breathlessness, wheezing, sleep disturbances, and variable airflow limitation (Reddel et al., 2015). These symptoms might lead to decreased physical activity and a sedentary lifestyle, which eventually further exacerbate asthma symptoms. The disease pathophysiology is associated with an overwhelming T helper cell type 2 (Th2) response, accompanied by high levels of immunoglobulin E (IgE) and Th2 cytokines [e.g., interleukin (IL)-4, IL-5, IL-9, and IL-13], and airway eosinophilia (Yazdanbakhsh et al., 2001). In particular, Th2 cytokine-induced mast cell activation results in the secretion of stored bronchoconstrictive mediators by granule exocytosis (Bradding et al., 2006). A plethora of inflammatory mediators might, in turn, fuel excessive reactive oxygen species (ROS) production, and diminished anti-oxidant defenses such as superoxide dismutase (SOD), catalase, and glutathione (GSH) (Sahiner et al., 2011). Disrupting the balance between oxidant and anti-oxidants may cause symptoms of chronic inflammation that characterize asthma, such as airway hyperresponsiveness (AHR), bronchoconstriction, mucus production, increased vascular permeability, and airway remodeling (Barnes, 1990; Nadeem et al., 2014). Thus, the reduction of both oxidative stress and inflammation can be a pivotal strategy for controlling asthma.

Aerobic exercise imparts anti-oxidant and anti-inflammatory effects that alleviate the symptoms of several diseases, including asthma (Brüggemann et al., 2015). Exercise assists in re-establishment of cellular homeostasis, reduction in levels of pro-inflammatory cytokines, and activation of the immune system (Ramos et al., 2010; Ávila et al., 2015; Weinhold et al., 2016; Lee et al., 2019). Proper aerobic exercise habits can reduce the likelihood of developing exercise-induced asthma by reducing ventilation during mild and moderate exercise, and it may reduce awareness of breathlessness via strengthening respiratory muscles (Chandratilleke et al., 2012). Exercise also enhances the levels of circulating catecholamines, such as epinephrine (Hewitt et al., 2010), which has been implicated in bronchodilatation (Gilbert et al., 1988). Aerobic exercise improves cardiovascular fitness and the quality of life of asthmatic patients by increasing their physical strength, neuromuscular coordination, and self-confidence (Chandratilleke et al., 2012).

Aquatic exercise (AE), such as swimming and walking in the water, is one of the safest forms of aerobic exercise and is the preferred rehabilitation therapy for asthmatics (Beggs et al., 2013) because of the lower exertional dyspnea, unlike running or cycling at the same intensity (Bar-Yishay et al., 1982). Ground-based exercise in cold, hot, or dry weather may worsen asthma symptoms. AE is performed in a humid and warm environment, which decreases the risk of exercise-induced asthma (Goodman and Hays, 2008). Further, the buoyancy of water reduces weight-bearing stress on the joints; therefore, relieving elderly patients from discomfort and distress of physical activity (PÖyhÖnen et al., 2002).

However, despite the undoubted positive advantages of exercise, incorrect exercise habits and practices could induce stress resulting in adverse consequences in terms of the immune function leading to immunosuppressive effects (Tuan et al., 2008). Besides, exercise-induced cytokines might increase the risk of infection (Gleeson, 2007), when exposed to aggressive environmental factors such as chlorinated swimming pools, sweat, and urine; thus, particular precautions involving immunosurveillance are required. Therefore, it has been suggested that persistence and reduction of immunity are dependent on the type, intensity, and duration of the exercise (Gleeson, 2007). Despite an emphasis on the importance of the type of exercise utilized, most studies have investigated the beneficial effects of AE without focusing on a particular exercise, and only a few studies have investigated the molecular mechanisms involved in asthma (Brüggemann et al., 2015).

This study was performed to investigate and compare the effects of different types of AE, walking and swimming, on an OVA-induced allergic airway inflammation in mice. In addition, we investigated the modulation of mitogen-activated protein kinase (MAPK)/NF-E2-related factor 2 (Nrf2)/heme oxygenase-1 (HO-1) and nuclear factor-κB (NF-κB) activations to identify the mechanism underlying the anti-oxidant and anti-inflammatory effects of AE allergic asthma.

## Materials and Methods

### Animals

Female BALB/c mice aged 5 weeks were obtained from Koatech Co. (Pyeongtaek, South Korea) and housed in a pathogen-free containment facility. Mice were provided with food and water *ad libitum*. Body weights of the mice were measured weekly for 4 weeks as well as on the day of operation. All animal treatments were approved by the Pusan National University-Institutional Animal Care and Use Committee in accordance with the National Institutes of Health Guidelines (PNU-2018-1827).

### Induction of Allergic Airway Inflammation

**FIGURE 1:**
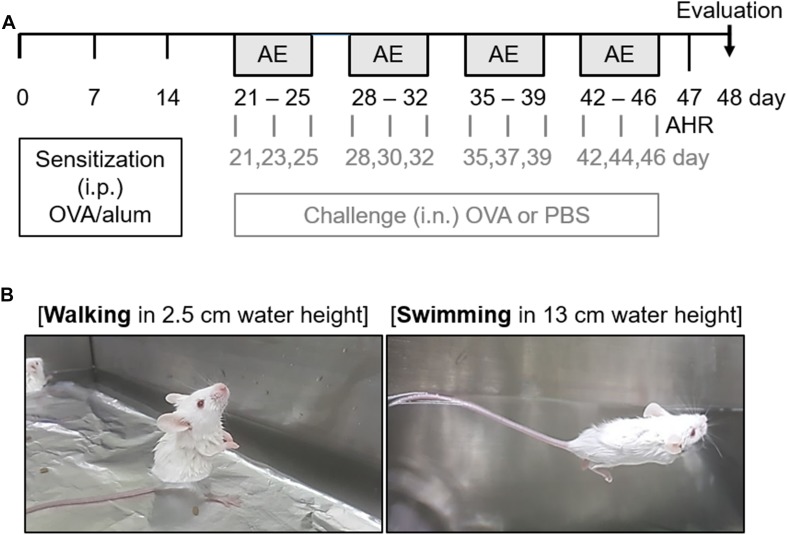
Experimental scheme of the aquatic exercise (AE) in a mouse model of allergic airway inflammation. **(A)** Mice were sensitized by an intraperitoneal (i.p.) injection of a mixture containing 100 μg of ovalbumin (OVA) and 2 mg of aluminum hydroxide (alum) in 0.2 mL of phosphate-buffered saline (PBS) on days 0, 7, and 14. Mice then received intranasal (i.n.) challenge with 50 μg of OVA in 50 μL of PBS, three times per week for 4 weeks. The control mice were challenged with PBS at the time of the OVA challenge. Twenty-four hours after the final challenge, lung function measurements were performed. All mice were sacrificed 48 h after the final challenge. **(B)** The different types of AE (walking and swimming) were performed in a water bath filled with water to a height of 2.5 and 13 cm for 30 min, respectively. The mice were made to swim for 4 s at a speed of 10 cm/s with 4-s intervals. The backs of the mice were tapped with a soft brush every 4 s to maintain the swimming intervals. This process was repeated five times a week for a total duration of 4 weeks.

Sensitization and challenge methods were modified based on a previous study (Ryu et al., 2017). As shown in Figure 1A, all mice were intraperitoneally sensitized by injection of 100 μg of OVA (Hyglos GmbH, Regensburg, Germany) dissolved in 200 μL phosphate-buffered saline (PBS) containing 2 mg of aluminum hydroxide (alum; InvivoGen, SanDiego, CA, United States) on days 0, 7, and 14. Mice were anesthetized with isoflurane (2% induction and 1.5% maintenance, in 80% N_2_O and 20% O_2_), and given intranasal challenge with 50 μg of OVA in PBS, three times per week, for 4 weeks. The control mice were sensitized and challenged with PBS at the time of OVA challenge. Twenty-four hours following completion of the last challenge, lung function measurements were taken. Two days after the last OVA challenge, all mice were sacrificed for analysis.

The mice were randomly divided into six groups (*n* = 8–11) as follows: challenge with PBS and non-exercise (Control); challenge with PBS and walking in water (Walking); challenge with PBS and swimming (Swimming); challenge with OVA and non-exercise (OVA); challenge with OVA and walking in water (OVA + Walking); and challenge with OVA and swimming (OVA + Swimming).

### Aquatic Exercise

One hour prior to each OVA challenge, two types of AE (walking and swimming) were performed in a constant-temperature water bath (width × length × height; 50 × 30 × 22 cm) filled with water to a height of 2.5 and 13 cm at 29°C for 30 min (Figure 1B). The mice were made to swim for 4 s at a speed of 10 cm/s with 4 s intervals. The backs of the mice were tapped with a soft brush every 4 s to maintain the swimming intervals. Following each exercise, the mice were gently dried with a towel. This process was repeated five times a week for a total duration of 4 weeks.

### Assessment of Airway Hyperresponsiveness

Airway Hyperresponsiveness was measured as the increase in pulmonary resistance after challenge with aerosolized methacholine (MCh) in conscious mice using the whole-body plethysmograph (OCP 3000, Allmedicus, Gyounggi, South Korea). Mice were challenged with various concentrations of methacholine (Sigma, St. Louis, MO, United States; 0, 12.5, 25, and 50 mg/mL in PBS) using a nebulizer (HARVARD73-1963, Harvard Apparatus, MA, United States) for 10 min at each concentration. The mice were immediately replaced in chambers, and the measurement was obtained 150 s after the completion of each nebulization. Enhanced pause (Penh) was automatically calculated based on the mean pressure generated in the plethysmography chamber.

### Collection of BALF and Cell Counting

Collection of Bronchoalveolar Lavage Fluid (BALF) and cell counts was performed as previously described (Ryu et al., 2017). To collect BALF, mice were sacrificed with a lethal dose of avertin tribromoethanol (Sigma), and the lung was lavaged with 1 mL ice-cold PBS via a tracheostomy tube. BALF was centrifuged at 300 × *g* for 10 min at 4°C, and total BAL cells were counted with a hemocytometer. BALF were frozen at −80°C for further cytokine analysis. Differential cells were attached to a slide by cytocentrifugation (Cytospin, Thermo Shandon, Pittsburgh, PA, United States) and stained with Diff-Quick (Sysmex International Reagents, Kobe, Japan). At least 300 cells were counted and classified as either macrophages, neutrophils, lymphocytes, or eosinophils.

### Enzyme-Linked Immunosorbent Assay

Serum concentration of IgE was determined using the mouse IgE ELISA kit (Bethyl Laboratories, Montgomery, TX, United States) and the OVA-specific IgE ELISA kit (Cayman Chemical, Ann Arbor, MI, United States). The levels of prostaglandin E_2_ (PGE_2_) and cytokines IL-4, IL-5, IL-13, IL-6, tumor necrosis factor (TNF)-α, tumor growth factor (TGF)-β1, and vascular endothelial growth factor (VEGF) were examined using their respective ELISA kits (R&D System, Minneapolis, MN, United States). Further tests included histamine (Enzo Life Sciences, Ann Arbor, MI, United States), mast cell tryptase (Cusabio Biotech., Wuhan, Hubei, China), and leukotrienes E4 (LTE4; MyBioSource, San Diego, CA, United States) in the BALF were also quantified using their respective ELISA kits. The absorbance was read at 450 nm using a microplate reader (Molecular Devices, Sunnyvale, CA, United States). The contents of MDA (Sigma, St. Louis, Mo. United States) and GSH (BioVision, Milpitas, CA, United States), and activity of SOD (BioVision, Milpitas, CA, United States) in lung tissues were measured using the ELISA kits.

### Lung Histology and Immunofluorescence

Following lavage, the lungs were fixed in 4% paraformaldehyde in PBS, embedded in paraffin, and cut into 5-μm sections. Sections were stained with hematoxylin and eosin (H&E; Sigma), periodic acid–Schiff (PAS; Millipore, Billerica, MA, United States), and Masson’s trichrome (MT; Polysciences, Warrington, PA, United States) to quantify the number of infiltrating inflammatory cells, mucus production, and collagen deposition by microscopy (BX-50 microscope, Olympus Co., Tokyo, Japan). The inflammation score of lung (H&E sections) was graded on the following scale: (0) no cells; (1) a few cells; (2) a ring of cells 1 cell layer-deep; (3) a ring of cells 2–4 cell layers-deep; and (4) a ring of cells > 4 cell layers-deep (Myou et al., 2003). The number of mucin-positive goblet cells (PAS sections) were graded on the following scale: (0) < 5% PAS-positive cells; (1) 5–25%; (2) 25–50%; (3) 50–75%; and (4) > 75% (Ford et al., 2001). The intensity of collagen deposition (MT sections) was graded on the following scale: (0) no collagen deposition; (1) a thin layer of collagen; (2) a cluster of collagen; and (3) a thick layer of collagen (Di Valentin et al., 2009).

To determine the thickness of the airway smooth muscle mass, after deparaffinization, lung sections were incubated overnight at 4°C with monoclonal anti-actin and anti-smooth muscle actin (α-SMA)-FITC antibody (1:500; Sigma). Area of α-SMA immunostaining was quantified to evaluate the thickness of the airway smooth muscle layer which was observed via a confocal laser scanning microscope (Olympus, Tokyo, Japan) for at least ten bronchioles of similar size from each group (Cho et al., 2004).

Histological assessment was performed by two independent observers in a blinded manner and results were indicated the average of observer’s scores.

### Western Blot

Lung tissues from experimental mice were lysed in RIPA lysis buffer (GenDEPOT, Barker, TX, United States) containing protease inhibitors; then total cellular proteins were isolated. Nuclear and cytosol proteins were extracted with a nuclear and cytosol protein extraction kit (Bio-world, Dublin, OH, United States). Equal amounts of protein samples were separated utilizing 10% sodium dodecyl sulfate-polyacrylamide gels and then electrotransferred onto nitrocellulose membranes (GE Healthcare Life Science, Piscataway, NJ, United States). Membranes were blocked with 5% skimmed milk, and incubated with specific primary antibodies at 4°C overnight: Nrf2 (1:1000; Abcam, Cambridge, MA, United States), HO-1 (1:1000; Enzo Life Sciences), p-ERK (1:2000; Cell Signaling, Danvers, MA, United States), ERK (1:1000; Cell Signaling), p-p38 (1:1000; Cell Signaling), p38 (1:1000; Cell Signaling), p-JNK (1:1000; Cell Signaling), and JNK (1:1000; Cell Signaling), and NF-κB (1:1000; Cell Signaling), were then washed three times and incubated with secondary antibody conjugated with horseradish peroxidase. Immunodetection was carried out with Western Lightning^®^ Plus-enhanced chemiluminescence kit (PerkinElmer, LAS, Inc., Waltham, MA, United States) and an Amersham Imager 680 (Fujifilm, Tokyo, Japan). Each band was quantitatively determined using ImageJ software (National Institute of Health). Membranes were stripped and incubated with β-actin (1:5000; Sigma) or Lamin B1 (1:1000; Cell Signaling) antibodies utilized as an internal control.

### Statistical Analysis

Data were expressed as the mean ± SEM. Comparisons among the experimental groups were created by one-way analysis of variance with Tukey’s *post hoc* test. Differences at *p* < 0.05 were determined to be statistically significant.

## Results

### Inhibition of OVA-Induced Airway Inflammation by AE

**FIGURE 2:**
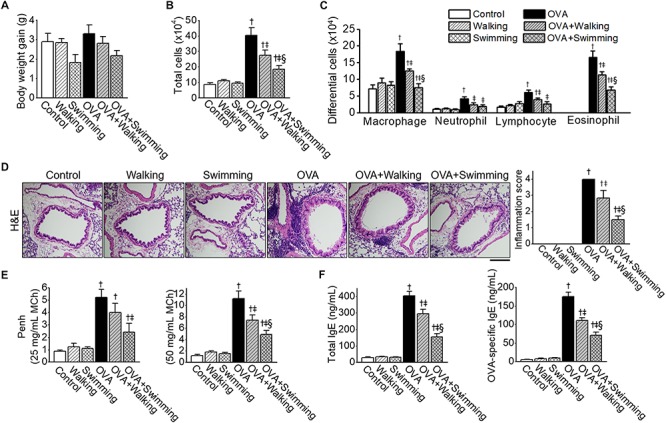
Inhibitory effects of Aquatic exercise (AE) on Ovalbumin (OVA)-induced airway inflammation. **(A)** Body weight gain of experimental groups. **(B)** Total cell counts in bronchoalveolar lavage fluid (BALF). Total cell counts were performed using a hemocytometer. **(C)** Differential cell counts in BALF were assessed by Diff-Quick staining. **(D)** Representative images of hematoxylin and eosin (H&E) staining of lung tissue. The scale bar represents 100 μm. The bar graphs depict the inflammation score (see section “Lung Histology and Immunofluorescence”). **(E)** Airway hyperresponsiveness (AHR) to methacholine challenge. **(F)** The levels of OVA-specific IgE in serum. Data are represented as the means ± SEM. ^†^*p* < 0.05, compared to control. ^‡^*p* < 0.05, compared to OVA. ^§^ *p* < 0.05, compared to OVA + Walking.

The body weight gain measured at the beginning and final days of the AE test was not statistically significant among experimental groups (Figure 2A).

The effects of different types of AE, walking and swimming, were examined on airway inflammation by OVA exposure, and inflammatory cells in the BALF were counted. Mice challenged with PBS, and AE (walking and swimming) failed to show a difference between the numbers of total and differential cells. However, an increase in the numbers of total cells from BALF in OVA mice was significantly reduced by both types of AE (Figure 2B); however, the swimming results showed a higher reduction than the walking mice. The numbers of macrophages, neutrophils, lymphocytes, or eosinophils in BALF were markedly increased in the OVA group, which was ameliorated by both types of AE (Figure 2C). Histological examinations revealed increases in inflammatory cells which infiltrated the peribronchial and perivascular regions of the lung in the OVA group (Figure 2D), these increases were reduced substantially by both AE. Specifically, lung inflammation was significantly lower in the OVA + Swimming group than in the OVA + Walking group.

To identify the effect of AE on OVA-induced AHR, methacholine challenges were performed. Elevated Penh values were observed in the OVA group at 25 and 50 mg/mL of MCh (Figure 2E), which were attenuated by AE. Penh values were significantly lower in the OVA + Swimming group than in the OVA + Walking group at the 50 mg/mL of MCh.

The levels of total and OVA-specific IgE in serum were compared among experimental groups. Both the total and OVA-specific IgE levels were elevated in the OVA group (Figure 2F), which was decreased by AE. Swimming showed better effects on lowering serum levels of IgE than walking.

### Reduction of OVA-Induced Elevated Th2 Cytokines, Leukotrienes, Histamine, and Mast Cell Tryptase Levels in BALF by AE

**FIGURE 3:**
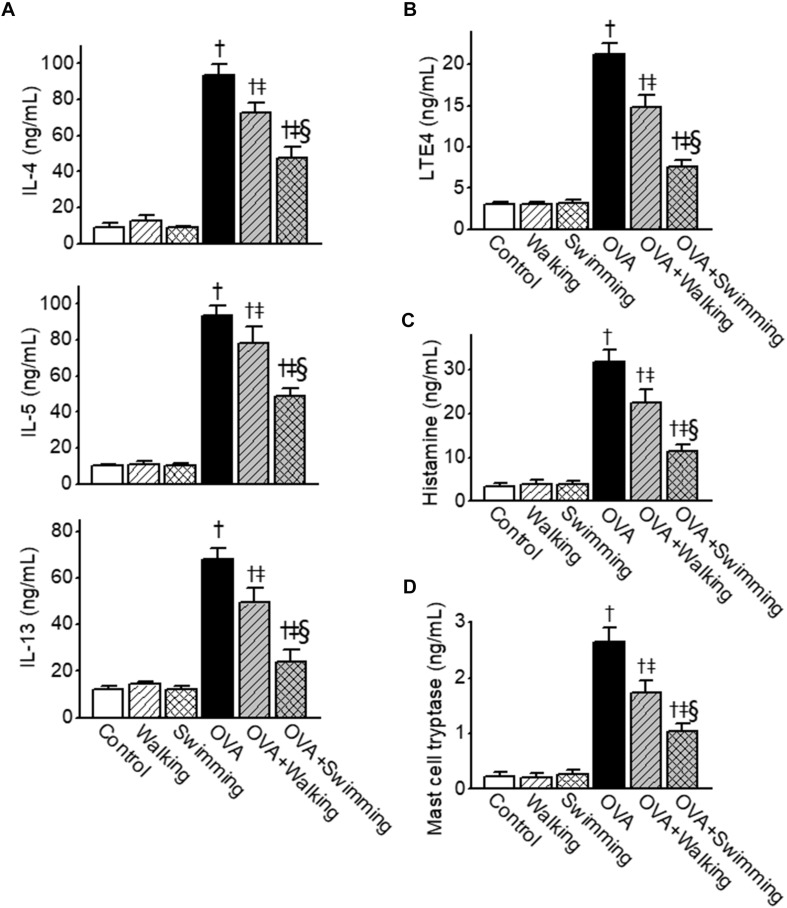
Inhibitory effects of Aquatic exercise (AE) on the ovalbumin (OVA)-induced increase in the levels of T helper cell type 2 (Th2)-derived cytokines, leukotriene E4 (LTE4), histamine, and mast cell tryptase in bronchoalveolar lavage fluid (BALF) obtained from mice. **(A)** Levels of interleukin (IL)-4, IL-5, and IL-13. **(B)** Levels of LTE4. **(C)** Levels of histamine. **(D)** Levels of mast cell tryptase. Data are represented as the means ± SEM. ^†^*p* < 0.05, compared to control. ^‡^*p* < 0.05, compared to OVA. ^§^ *p* < 0.05, compared to OVA + Walking.

To determine the effects of AE on Th2 cytokine release in OVA-challenged mice, levels of IL-4, IL-5, and IL-13 in BALF were measured. IL-4, IL-5, and IL-13 levels in BALF were markedly increased in the OVA-challenged mice (Figure 3A). In the AE groups, Th2 cytokine elevation was considerably suppressed compared to that in the OVA group. Concentrations of LTE4, histamine, and mast cell tryptase (all bronchoconstriction mediators) were examined in BALF from the experimental group. Increases in the levels of LTE4, histamine, and mast cell tryptase in OVA group were significantly attenuated by AE (Figures 3B–D). Inhibitory effects of swimming were significantly higher than walking on OVA-induced expression of Th2 cytokines and bronchoconstrictive mediators.

### Inhibitory Effect of AE on OVA-Induced Airway Remodeling

**FIGURE 4:**
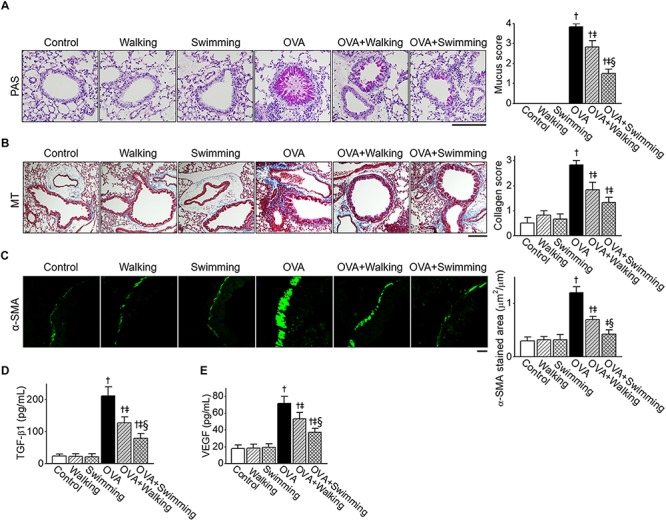
Reduction of ovalbumin (OVA)-induced airway remodeling by Aquatic exercise (AE). **(A)** Representative images of periodic acid-Schiff (PAS) staining of goblet cells in the experimental groups. The scale bar represents 100 μm. The bar graphs depict the number of PAS-positive mucus-producing cells (see section “Lung Histology and Immunofluorescence”). **(B)** Representative images of Masson’s trichrome (MT) staining of peribronchial areas. The scale bar represents 100 μm. The bar graphs depict the collagen deposition score (see section “Lung Histology and Immunofluorescence”). **(C)** Representative α-smooth muscle actin (α-SMA) and FITC expression determined by immunohistochemistry of similar size bronchioles. The scale bar represents 20 μm. The bar graphs depict the area of α-SMA staining per micrometer length of the bronchiolar basement membrane (μm^2^/μm). **(D)** Level of tumor growth factor (TGF)-β1 in lung tissues. **(E)** Level of vascular endothelial growth factor (VEGF) in lung tissues. Data are represented as the means ± SEM. ^†^*p* < 0.05, compared to control. ^‡^*p* < 0.05, compared to OVA. ^§^*p* < 0.05, compared to OVA + Walking.

We examined features of airway remodeling in lung tissues by determining whether AE impacted OVA-induced airway remodeling. Hyperplasia of mucus-secreting goblet cells in lung tissues was observed using PAS staining (Figure 4A). Increased mucus production was detected around the peribronchial area of the OVA group. However, AE markedly decreased overproduction of mucus by OVA. Collagen deposition in lung tissues was observed using MT staining (Figure 4B). Further, the increased fibrotic area in the OVA group was alleviated by AE. Inhibitory effects of U-AE on collagen deposition was higher than that of P-AE, but not statistically significant between the OVA + Walking and OVA + Swimming. The airway smooth muscle mass was analyzed by immunostaining with α-SMA. As depicted in Figure 4C, the increased airway smooth muscle mass in the OVA group was significantly alleviated by AE, indicating reduced airway remodeling. The improvement effect of swimming on OVA-induced airway remodeling was also higher than that of the walking. Next, the levels of airway remodeling-related cytokines TGF-β1 and VEGF were determined from lung tissues. The levels of TGF-β1 and VEGF markedly increased in the OVA group and were profoundly decreased by AE (Figures 4D,E). In particular, swimming showed higher inhibitory effects on the levels of TGF-β1 than walking.

### Anti-oxidant Effects of AE Through Modulation of MAPK/Nrf2/HO-1

**FIGURE 5:**
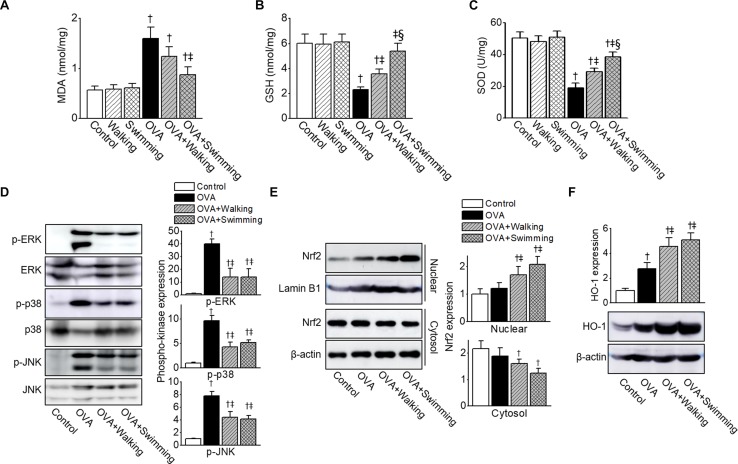
Inhibition of ovalbumin (OVA)-induced oxidative stress by Aquatic exercise (AE). **(A)** Contents of malondialdehyde (MDA), **(B)** glutathione (GSH), and **(C)** superoxide dismutase (SOD) activity in the lung tissues. **(D)** Reduction in levels of phosphorylated p-ERK, p-p38, and p-JNK by AE. The total protein isolated from lung tissues was subjected to Western blot analysis using anti-p-ERK, anti-ERK, anti-p-p38, anti-p38, anti-p-JNK, and anti-JNK antibodies. **(E)** Representative bands of NF-E2-related factor 2 (Nrf2) in the nuclear and cytosol, as examined by western blot analysis in lung tissues. The bar graphs depict a relative density of immuno-positive bands. The anti-Lamin B1 or anti-β-actin antibodies were used as an internal control. **(F)** Representative protein bands of heme oxygenase-1 (HO-1), as determined by western blot analysis in lung tissues. The bar graphs depict a relative density of immuno-positive bands. The anti-β-actin antibody was used as an internal control. The bar graphs depict a relative density of immuno-positive bands. Data are represented as the means ± SEM. ^†^*p* < 0.05, compared to control. ^‡^*p* < 0.05, compared to OVA. ^§^ *p* < 0.05, compared to OVA + Walking.

To investigate whether AE modulated OVA-induced oxidative injury, oxidative stress markers MDA, GSH, and SOD were measured in the lung tissues. OVA-challenged mice showed significantly increased MDA and decreased GSH levels in the lung tissues compared to those of the control group mice, and AE significantly decreased MDA and increased GSH production (Figures 5A,B). The activity of SOD in the lung was reduced in the OVA group, whereas AE upregulated SOD activity (Figure 5C). The swimming showed higher anti-oxidant activities in OVA-challenged mice than those of walking. Both types of AE failed to evoke effects on MDA, GSH, and SOD in the control group.

Increased ROS leads to activation of MAPK pathways. To determine whether AE regulated the MAPK pathways, we examined MAPK activation in lung tissues obtained from experimental groups. As shown in Figure 5D, the phosphorylation of ERK, p38, and JNK was upregulated in the OVA-challenged mice. AE markedly inhibited phosphorylation of MAPKs by OVA.

The Nrf2/HO-1 pathway is one of the most important mechanisms of the cellular defense system. To verify whether AE activated the Nrf2/HO-1 pathway in allergic airway inflammation, we determined expression levels of Nrf2 and HO-1 in the lung tissues obtained from mice. The expression levels of Nrf2 and HO-1 were higher in the OVA group than in the control group (Figures 5E,F), which was further upregulated in AE groups.

### Anti-inflammatory Effects of AE Through Inactivation of NF-κB

**FIGURE 6:**
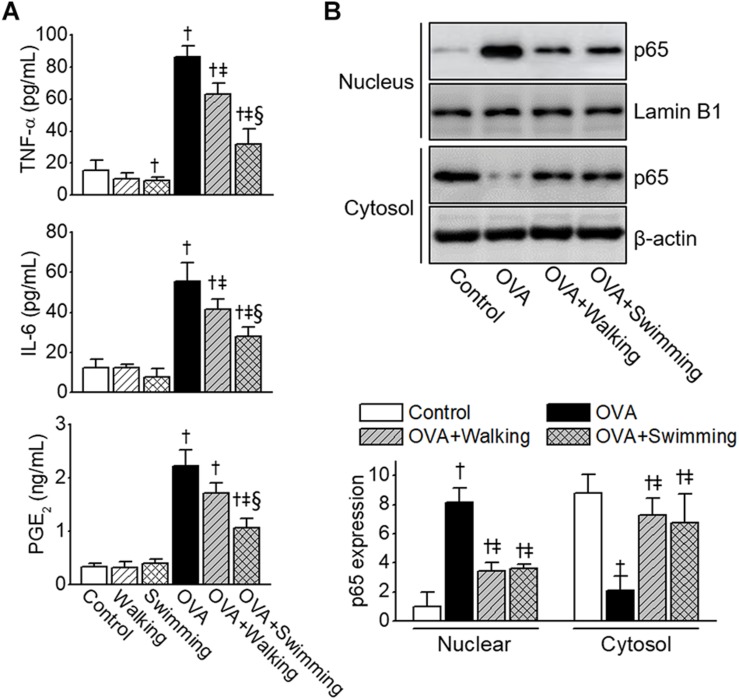
Reduction of ovalbumin (OVA)-induced airway inflammation by Aquatic exercise (AE). **(A)** Levels of tumor necrosis factor (TNF)-α, IL-6, and prostaglandin E_2_ (PGE_2_) in bronchoalveolar lavage fluid (BALF). **(B)** Inhibition of OVA-induced NF-κB nuclear translocation by AE. Nuclear and cytoplasmic fractions were extracted and subjected to Western blot analysis using the anti-NF-κB p65 antibody. The anti-Lamin B1 or anti-β-actin antibodies were used as an internal control. The bar graphs depict a relative density of immuno-positive bands. Data are represented as the means ± SEM. ^†^*p* < 0.05, compared to control. ^‡^*p* < 0.05, compared to OVA. ^§^ *p* < 0.05, compared to OVA + Walking.

To analyze the effects of AE on the production of pro-inflammatory cytokines and potent inflammatory mediators in OVA-induced allergic airway inflammation, levels of TNF-α, IL-6, and PGE_2_ in BALF were analyzed. The levels of TNF-α, IL-6, and PGE_2_ in the OVA group were found to be significantly elevated compared to those of control group (Figure 6A). However, these levels were attenuated by AE, especially by swimming, resulting in a significant reduction compared to those of the walking group. As NF-κB plays a pivotal role in pathogenesis of the allergic inflammatory response, we examined the activation of NF-κB in lung tissues obtained from experimental groups. As depicted in Figure 6B, the OVA-induced significant NF-κB activation and cytoplasm-to-nucleus translocation were reduced by AE.

## Discussion

To our knowledge, this is the first study to demonstrate the inhibitory effects of different types of AE focusing on resistance, walking and swimming, in a mouse-based model of OVA-induced allergic airway inflammation. The results showed multiple inhibitory effects caused by aerobic exercise imparted to asthma-related responses including the reduction of inflammatory cell infiltration, AHR, levels of serum IgE, concentrations of Th2 cytokines, LTE4, histamine, and tryptase in BALF. Penh is not a reliable measurement to assess airway resistance; however, we indirectly present the Penh value to inhaled MCh as a parameter reflecting function of lower airway because of limitations of instruments to assess airway resistance. AE also significantly prevented features of airway remodeling including mucus production, collagen deposition, and thickness of the smooth muscle, lung TGF- β1, and VEGF levels. AE increase levels of both GSH and SOD and decrease MDA contents. AE also reduced the release of TNF-α, IL-6, and PGE_2_ in BALF. Water depth appeared to be a factor, as AE at water height of 13 cm resulted in greater effects than walking at water height of 2.5 cm, including a significant reduction in airway inflammation. These effects most likely through inducing activation of Nrf2/HO-1 and inhibition of MAPK/NF-κB signaling.

Numerous studies have reported the beneficial effects of AE on pulmonary diseases. High-intensity AE over 3 weeks showed anti-oxidant and anti-inflammatory effects in OVA-induced allergic asthma (Brüggemann et al., 2015) and lipopolysaccharide (LPS)-induced non-allergic asthma in mice (Cardoso et al., 2018). High-intensity AE decreased the number of inflammatory cells and release of IL-1β, IL-6, and TNF-α levels and increased the levels of IL-10, IL1ra, and catalase activity in diesel exhaust particles (DEP)-induced pulmonary inflammation in mice (Ávila et al., 2015). Other studies have reported that low-intensity AE attenuated lung neutrophilic inflammation in LPS-treated acute lung injury mice (Ramos et al., 2010).

Currently, the interrelationships between the potential risks of chlorinated swimming pools and the prevalence of asthma during AE are being investigated. Most swimming pools worldwide are disinfected by the chlorination method, with the resulting changes leading to the production of by-products, which are present at high levels on the water surface, causing irritation of the skin and mucosa of the respiratory tract (Chowdhury et al., 2014). Further, not only chronic exposure to low dose but also acute exposure to high dose chlorine induces airway inflammation and AHR in mice (Martin et al., 2003; Kim et al., 2014). Exposure to a chlorinated pool contributes significantly to the development of asthma and respiratory allergies in adolescents (Bernard et al., 2009). Competitive swimmers, indoor swimming pool workers, and cleaners, who are frequently exposed to the chlorine environment, are particularly concerned about the increase in occupational asthma (Varraso et al., 2002; Medina-Ramon et al., 2005; Jacobs et al., 2007). Moreover, while the negative effects of high levels of chlorine on the pulmonary system are well-known, the effects of daily low-level exposure to these compounds have not been fully explicated explained.

Th2 cytokines are considered key players in the pathogenesis of asthma, because they promote infiltration of eosinophils into the airways, AHR, mucus hypersecretion, smooth muscle hyperplasia, IgE production, and mast cell activation (Barnes, 2001; Kay et al., 2004). Further, LTE4, histamine, and tryptase released from IgE and activated mast cells induced maturation, functional activation, and migration of dendritic cells (Galli and Tsai, 2012), leading to increased airway smooth muscle contractility and bronchoconstriction (Berger et al., 2001). Further, bronchoconstriction mediators LTC4, LTD4, and LTE4 are closely related to asthma phenotypes (Scott and Peters-Golden, 2013), and their antagonists are a useful target for asthma therapies (Israel et al., 2002; Tantisira et al., 2009). Moderate-intensity aerobic exercise inhibits OVA-induced asthmatic parameters by modulation of LT signaling (Alberca-Custódio et al., 2016). These pathophysiological changes were confirmed in the OVA-challenged mice in our study, which were attenuated by both walking and swimming.

Airway remodeling, which is correlated with the severity of asthma (Pascual and Peters, 2005), is characterized by goblet cell and submucosal gland enlargement, subepithelial fibrosis, and increased smooth muscle mass (Xu et al., 2011). Our study showed that AE could reverse asthma-induced features of airway remodeling as well as levels of pro-fibrotic cytokine TGF-β1 and vasculogenesis-related growth factor VEGF in the lungs. These results strongly indicated that AE could serve as a novel therapy to target airway remodeling and provide additional benefits for asthma management.

Oxidative stress is the result of the imbalance between oxidant and anti-oxidant in favor of the oxidant, ROS generated under oxidative stress induces biomolecular damage and inflammation. To prevent uncontrolled ROS formation several defense systems are involved in the organism. These systems that include non-enzymatic molecules such as GSH as well as enzymatic scavengers of ROS, with SOD are the best-known defense systems. GSH and SOD are one of the most central physiological anti-oxidants against free radicals and they prevent subsequent lipid peroxidation (Suzer et al., 2000). Lipid peroxidation is a process generated naturally in small amounts in the body, mainly by the effect of several ROS and is the most common consequence of oxidative damage. MDA is one of the end-product of lipid peroxidation and is toxic to cells and cell membranes.

Oxidative stress is regarded as an inducer of airway inflammation; thus, it is a key factor of asthma progression (Riedl and Nel, 2008). In asthma, ROS is generated either directly from infiltrating inflammatory cells (e.g., eosinophils, mast cells, neutrophils, and T lymphocytes) or through the production of lipid peroxidation such as MDA (Fatani, 2014). It also involved in pathophysiological changes of asthma, such as reactivity and secretions of airway, vascular permeability, and increases of lipid peroxidation (Fatani, 2014). In this study, OVA group showed significant increases in the MDA levels, which were inhibited by both AE. In particular, swimming showed higher inhibitory effects on the levels of MDA than walking. Besides, reduction of GSH levels and SOD activities due to OVA was significantly increased by both AE. Specifically, the rise effects of GSH levels and SOD activities were significantly higher in the OVA + Swimming group than in the OVA + Walking group.

Mitogen-activated protein kinase activation eventually results in inflammatory gene transcription and pro-inflammatory cytokine and chemokine induction. MAPK signaling is important in the pathogenesis of immune, inflammation, and remodeling events occurring during asthma (Blease, 2005). This has been demonstrated in studies showing inhibition of antigen-induced asthma by U0126 (ERK inhibitor) (Duan et al., 2004), SB239063 (p38 inhibitor) (Underwood et al., 2000), or PD98059 (JNK inhibitor) (Eynott et al., 2004) in mouse models of allergic airway inflammation. MAPK activation markedly increases in lung tissues obtained in OVA-challenged mice. However, AE significantly inhibited MAPK signaling pathway. Further, AE markedly enhanced Nrf2/HO-1 signaling activity compared to that in the OVA group. Nrf2 is an important transcription factor regulating the expression of phase II defense enzymes including HO-1 via anti-oxidant response elements (ARE) (Alam and Cook, 2003). Nrf2 is sequestered in the cytoplasm in combination with repressor molecules, Kelch-like ECH-associated protein 1 under normal physiological conditions. However, upon the appropriate stimulation, it translocates to the nucleus where it binds to the ARE and induced anti-oxidant enzyme, to offset oxidative stress (Jaiswal, 2004; Kobayashi and Yamamoto, 2005). Therefore, AE possesses anti-oxidant effects in OVA-induced asthma by promoting the inhibition of MAPK and activation of Nrf2/HO-1 signaling.

Activation of NF-κB signaling eventually resulted in inflammatory gene transcription and pro-inflammatory cytokines and chemokine induction. NF-κB is involved in various inflammatory networks modulating cytokine activity in airway pathology. One of the most important results of this study was that AE significantly reduced the levels of TNF-α, IL-6, and PGE_2_ through suppressing NF-κB pathways.

Previous studies demonstrated that a single bout of moderate-intensity aerobic exercise decreased airway inflammation via inhibition of NF-κB phosphorylation, but not AHR or airway remodeling in OVA-challenged mice (Hewitt et al., 2009). While, repeated bouts of moderate-intensity aerobic exercise decreased AHR, airway smooth muscle thickness and increased levels of circulating epinephrine through a mechanism that involves β2-adrenergic receptors (Hewitt et al., 2010). Long-term treadmill aerobic exercise inhibited DEP-induced lung inflammation, oxidative, and nitrosative stress in mice (Vieira et al., 2012). Aerobic exercise reduced OVA-induced airway inflammatory responses via inhibition of NF-κB activation in atopic mice (Pastva et al., 2004). Treadmill aerobic exercise inhibited airway inflammation and remodeling in OVA-treated guinea pigs (Olivo et al., 2012). Two intensity, low- and moderate-treadmill aerobic exercise showed remarkable reductions in eosinophilic lung inflammation and airway remodeling, which was increased by OVA, with similar effects to that of low- or moderate- aerobic exercise in OVA-challenged mice (Vieira et al., 2007). High-intensity AE, decreased severity and pathology of experimental autoimmune encephalomyelitis in mice (Xie et al., 2019). Conflicting results from these studies might be due to the use of subjective criteria without scientific exactitude for the method, intensity, and duration of aerobic exercises. In our results, the anti-asthmatic effects of swimming were found to be greater than that of walking in OVA-challenged mice.

## Conclusion

Our study provides evidence that AE effectively reduces the levels of biomarkers related to oxidative stress and inflammation, the two pathways involved in allergic airway inflammation mice. Moreover, this study found that the beneficial effects of AE might be associated with Nrf2/HO-1 defense pathway activation and MAPK/NF-κB pathway suppression. Asthmatics who have difficulty in swimming may gain a beneficial effect by merely walking in non-chlorinated swimming pools at a high humidity and warm air temperature; thus, it is our belief that AE prescription should be part of the therapy for allergic asthma.

## Data Availability Statement

The datasets generated for this study are available on request to the corresponding author.

## Ethics Statement

The animal study was reviewed and approved by Pusan National University-Institutional Animal Care and Use Committee in accordance with the National Institutes of Health Guidelines (PNU-2018-1827).

## Author contributions

BL and JR designed and performed the experiments, and wrote the manuscript with all authors contributing to writing. YK, YMK, JJ, TK, and S-YL analyzed and interpreted the data. Y-IS supervised the study and reviewed the manuscript.

## Conflict of Interest

The authors declare that the research was conducted in the absence of any commercial or financial relationships that could be construed as a potential conflict of interest.
